# Tetraparesis and sensorimotor axonal polyneuropathy due to co-occurrence of Pompe disease and hereditary ATTR amyloidosis

**DOI:** 10.1007/s10072-020-04896-3

**Published:** 2020-11-13

**Authors:** Milan Zimmermann, Natalie Deininger, Sophia Willikens, Tobias B. Haack, Kathrin Grundmann-Hauser, Berthold Streubel, Melanie Schreiber, Holger Lerche, Alexander Grimm

**Affiliations:** 1 Department of Neurology and Hertie Institute for Clinical Brain Research and Center of Neurology, University of Tübingen, Hoppe-Seyler-Str. 3, 72076 Tübingen, Germany; 2 German Center for Neurodegenerative Diseases (DZNE), 72076 Tübingen, Germany; 3 Department of Neurodegenerative Diseases, University of Tübingen, Hoppe-Seyler-Str. 3, 72076 Tübingen, Germany; 4 Institute of Medical Genetics and Applied Genomics, University of Tübingen, 72076 Tübingen, Germany; 5 Center for Rare Diseases, University of Tübingen, Tübingen, 72076 Tübingen, Germany; 6 Institute for Pathology, Medical University of Wien, 1090 Wien, Austria

**Keywords:** Pompe disease, Amyloidosis, Polyneuropathy, Transthyretin, Axonal degeneration

## Abstract

**Introduction/aims:**

Hereditary transthyretin amyloidosis with polyneuropathy (hATTRPN) is an autosomal dominant multi-organ disorder manifesting in the third to fifth decade with the key clinical features of distal and painful sensory loss of the lower limbs and autonomic dysregulation. Motor neuropathy and cardiomyopathy evolve in the course of the disease. Pompe disease is an autosomal recessive disease leading to decreased levels of lysosomal enzyme acid α-glucosidase and proximal muscle weakness. We report the clinical features and diagnostic workup in the rare case of a patient with ATTR amyloidosis and late-onset Pompe disease, both genetically confirmed.

**Methods:**

We performed a detailed clinical assessment, exome sequencing, and biochemical measurements.

**Results:**

The patient presented with a distal, painful hypaesthesia of both legs, a cardiomyopathy, and a muscle weakness in the form of a girdle-type pattern of the arms and legs at the beginning and a spreading to distal muscle groups in the course of disease.

**Discussion:**

This study highlights the importance of searching for co-occurrence of rare monogenetic neuromuscular diseases, especially in cases in which all clinical features can be readily explained by a single gene defect.

**Supplementary Information:**

The online version contains supplementary material available at 10.1007/s10072-020-04896-3.

## Introduction

Hereditary transthyretin amyloidosis with polyneuropathy (hATTR-PN) (OMIM 105210) is an autosomal dominant disorder mostly beginning in the third to fifth decade with a distal sensory loss of the lower limbs, accompanied by burning feet, predominant vegetative dysregulation, and also a distal predominant motor neuropathy over the course of the disease. Electrophysiological examinations in endemic countries like Portugal often point towards a length-dependent small-fiber polyneuropathy [2].

A therapeutic option is the oral treatment with tafamidis, which stabilizes the tetramer structure of transthyretin preventing the amyloid deposition.

Pompe disease is an autosomal recessive disorder which results from a deficiency of the lysosomal enzyme acid α-glucosidase leading to an accumulation of lysosomal glycogen in the muscle tissue of smooth and skeletal muscle. Adults mostly present with a proximal muscle weakness with the pelvic girdle being more often affected than the shoulder girdle or an axial myopathy. An involvement of the intercostal muscles might result into respiratory insufficiency. There might also be a rigid spine syndrome. Elevated muscle enzyme creatinkinase might be one hallmark [3].

In extension of the classical phenotypes, we here report on a patient with severe distally stressed tetraparesis and sensorimotor polyneuropathy resulting from a combined defect of lysosomal enzyme acid α-glucosidase (Pompe disease) and hATTR-PN.

## Methods

Exome sequencing was performed on patient’s genomic DNA. Exonic and adjacent intronic regions were enriched using a SureSelectXT Human All Exon v7 kit (Agilent Technologies, Santa Clara, CA). Sequencing as 2 × 100 bp paired-end reads was performed on a NovaSeq6000 (Illumina, San Diego, CA) to an average 170.5X coverage. Generated sequences were analyzed using the megSAP pipeline (https://github.com/imgag/megSAP). We next prioritized rare (MAF < 0.1% in 1000 genomes, ExAC or GnomAD, in-house database) nonsynonymous DNA variants in genes that have been associated with the patient’s clinical features.

Testing for alpha-1,4 glucosidase activity was performed from dried blood spot using a validated and accredited tandem mass spectrometry method [6]. Test was performed at the medical laboratories of ARCHIMED Life Science GmbH (www.archimedlife.com).

### Case report

A 62-year-old male patient presented with a rapidly progressive gait disorder. The symptoms started at an age of 57 years with a hypoesthesia and paresthesia of the toes on both sides. Half a year later, he developed a left-pronounced hypoesthesia of both legs with frequent attacks of electric shock-like pain. At the age of 58 years, he started to experience a distal muscle weakness of the left leg and fasciculation of the lower extremities with inability to stand on his toes at the age of 59 years. At the age of about 62 years, a wheelchair-dependency developed. The motoric symptoms comprised a distally stressed paresis of the upper extremities in the course of disease. The examination at the age of 62 years revealed decreased grades of muscle strength according to Janda of both upper and lower extremities. At the first examination at the age of 58 years, there was no involvement of the arms and a more proximally and left stressed reduction of muscle strength. All tendon reflexes could not be triggered anymore, and he showed severe distally stressed atrophies. The patient also presented with a hypoesthesia of both forearms and both legs with inability to recognize temperature differences in the lower legs. He also described urinary urgency. There were no clinical signs of dyspnea, dysphagia, constipation, hypersalivation, erectile dysfunction, or hyperhidrosis.

After diagnosis of hATTR-PN and a Pompe disease at the age of 62 years, a combined therapy with tafamidis (20 mg/day) and alglucosidase alfa (1250 mg every 2 weeks) was initiated. This led to a significant reduction of hypoesthesia and the shock-like pain in the lower limbs and increase in muscle strength in the course of 3 months. Additionally, he was better able to raise up from bed by using his arms. After about 6 months, he could stop the former opioid-based pain-reducing medication. However, the patient experienced an increasing cold feeling of hands and feet.

Electrophysiological examinations revealed sensorimotor axonal polyneuropathy (see supplemental table 1). The electromyography (EMG) showed pathological spontaneous activity in both proximal and distal muscles before begin with treatment with alglucosidase alpha (M. biceps brachii, M. abductor digiti minimi, and M. vastus lateralis on the right side and M. tibialis anterior on the left side). The EMG depicted a reduced interference pattern initially. In a control examination after 6 months (or 3 months after begin with treatment with alglucosidase alpha), a dense interference pattern was found. Transthoracic echocardiography showed hypertrophia of the left ventricle with moderately restricted pump function being compatible with a cardiac amyloidosis. A MR tomography of the spine showed compression of the right-sided C6 nerve root and the left-sided C7, L3, and L4 nerve roots due to uncovertebral arthrosis and herniated discs. The blood analysis revealed an increase in creatinkinase of up to 323 U/l (norm < 190 U/l).

His 89-year-old mother also had a progressive gait disorder due to a distally stressed polyneuropathy of both legs starting at the age of 81 years. His 55-year-old sister and his 65-year-old brother were not affected by a polyneuropathy. A genetic analysis was not performed yet.

The genetic analysis identified a heterozygous missense variant in *TTR* (ENST00000237014, c.379A>G, p.Ile127Val) as well as two heterozygous changes in *GAA* (ENST00000302262, ENST00000302262, c.-32-13T>G(;)1478C>T, p.(?)(;)(Pro493Leu)). All changes are absent from gnomAD or have a very low frequency (MAF 0.0029 gnomAD control population for c.-32-13T>G). These variants are annotated as pathogenic in ClinVar and HGMD and have been reported as clinically relevant in several unrelated individuals with hATTR-PN (*TTR*, OMIM #105210) [4], or glycogen storage disease II [5] (*GAA*, OMIM #232300).

A deficiency of the GAA protein was subsequently confirmed by functional studies showing a reduced glucosidase activity (0.3 μmol/l/h (normal > 2.0 μmol/l/h)).

## Discussion

We present a patient with mutations leading to a deficiency of lysosomal enzyme acid α-glucosidase (Pompe disease) and hATTR-PN. The patient reported the typical clinical course of hATTR-PN with hypoesthesia of both feet at the beginning and progression of the disease with involvement of proximal lower limbs and at least also of the upper limbs and frequent attacks of electric shock-like pain. He also showed the typical signs of a dysfunction and hypertrophy of the left ventricle. The motor signs especially affected the proximal muscle groups with left and proximally stressed muscle weakness, atrophies, and fasciculation in the beginning, as it is typical for late-onset Pompe disease [3], but not for the mostly distally stressed ATTR amyloidosis [2]. Interestingly, the pattern of muscle weakness shifted from an involvement of especially proximal localized muscles at the beginning to a distally stressed muscle disease with inability to move both feet in the course of disease. An axial myopathy, which is frequently described in patients with Pompe disease, was not present. The electromyography revealed pathological spontaneous activity in both proximal and distal localized muscles. In contrast to this, recent findings revealed especially an involvement of proximal muscles in patients with Pompe disease [7]. Especially the follow-up examination depicting a dense interference pattern is compatible with the diagnosis of a myopathy, whereas the first examination showed a reduced interference pattern.

This is to our knowledge the first description of a co-occurrence of both genetic diseases. Abeykoon et al. presented a case report about a 70-year-old woman with dysphagia and dysarthria due to a macroglossia. She also had a proximal muscle weakness. The diagnostic evaluation revealed the co-occurrence of the age-related wild-type transthyretin amyloidosis and Pompe disease [1].

This novel phenotype of our patient might be due to the interaction of both diseases leading to an impairment of muscle tissue by two different pathways, first by glycogen accumulation and second by axonal damage of motor nerves.

Our study highlights the need for recognition of co-occurrence of different genetic diseases, especially if there is not a comprehensive explanation for all of the cardinal symptoms. This might have important impacts for the treatment of patients as our case report has shown.

## Supplementary Information

ESM 1 (DOCX 21 kb)
