# Supplementary material for: Tetraparesis and sensorimotor axonal polyneuropathy due to co-occurrence of Pompe disease and hereditary ATTR amyloidosis
Source: Neurol Sci. 2020 Nov 13;42(4):1523–5. doi: 10.1007/s10072-020-04896-3 (PMC7955998; doi:10.1007/s10072-020-04896-3)
Supplement: Supplementary file 1 — (DOCX 21 kb) [file 10072_2020_4896_MOESM1_ESM.docx]

# **Supplementary table 1:**

## **Nerve conduction studies**

Nerve conduction findings are given with normal reference values of the respective lab in parenthesis. In case more numbers are given, these represent a disal/proximal stimulation.

dL: distal motor latency. CMAP: Compound muscle action potential. mNCV: motor nerve conduction velocity. F: minimal F-wave latency. SNAP: Sensory nerve action potential. SNCV: Sensory nerve conduction velocity. L: Left side. R: right side. TP: time point. NP: No potential

|  | **TP 1** | **TP 2 (after 2 month)** | **TP 3 (after 3 years)** | **TP 3 (after 4 years and 3 month after begin with tafamidis and alglucosidase alfa)** |
| --- | --- | --- | --- | --- |
|  | Age 58 years | Age 58 years | Age 61 years | Age 63 years |
| Tibial dL [ms]  CAMP [mV]  mNCV [m/s] | 6.0 (R) (<5.1)  1.1/1.1 (R) (>5.0)  46.0 (R) (>40) | 4.7 (R) (<5.1)  1.5/1.4 (R) (>5.0)  38.0 (R) (>40) |  | NP (R) |
| Ulnar dL [ms]  CAMP [mV]  mNCV [m/s] | 3.0 (L) (<3.2)  17.0/15.5 (L) (>4.0)  65.0 (L) (>50) | 3.1 (R) (<3.2)  19.8/18.9 (R) (>4.0)  52 (R) (>50) | 3.3 (R) (<3.2)  6.8/5.1/3.7 (R) (>4.0)  52.5/38.9 (R) (>50) | 3.8 (L) (<3.2)  8.1/7.4 (L) (>4.0)  56 (L) (>50) |
| Median dL [ms]  CAMP [mV]  mNCV [m/s] |  |  | 7.1 (R) (<4.6)  2.2/1.9 (R) (>5.0)  45 (R) (>50) |  |
| Peroneus dL [ms]  CAMP [mV]  mNCV [m/s] | 5.6 (L)  0.5/0.5 (L) (>4.0)  41.0 (L) (>41) | 8.5 (L)  0.55/0.5 (L) (>4.0)  35.0 (L) (>41) |  | NP (L) |
| Sural  SNAP [µV]  SNCV [m/s] | NP (R/L) | NP (R/L) |  | NP (R) |
| Ulnar  SNAP [µV]  SNCV [m/s] | NP (R) | 4.5 (R) (>5.8)  46 (R) (>44) | NP (R) | 1.6 (R) (>5.8)  41 (R) (>44) |
| Median  SNAP [µV]  SNCV [m/s] |  |  | NP (R) |  |
| Radialis  SNAP [µV]  SNCV [m/s] |  |  | NP (R) |  |
